# A Review on the Applications of Next Generation Sequencing Technologies as Applied to Food-Related Microbiome Studies

**DOI:** 10.3389/fmicb.2017.01829

**Published:** 2017-09-21

**Authors:** Yu Cao, Séamus Fanning, Sinéad Proos, Kieran Jordan, Shabarinath Srikumar

**Affiliations:** ^1^UCD-Centre for Food Safety, Science Centre South, University College Dublin Dublin, Ireland; ^2^Food for Health Ireland, Science Centre South, University College Dublin Dublin, Ireland; ^3^Teagasc, Food Research Centre Fermoy, Ireland

**Keywords:** next generation sequencing, food microbiome, 16S rDNA, metagenomics, metatranscriptomics

## Abstract

The development of next generation sequencing (NGS) techniques has enabled researchers to study and understand the world of microorganisms from broader and deeper perspectives. The contemporary advances in DNA sequencing technologies have not only enabled finer characterization of bacterial genomes but also provided deeper taxonomic identification of complex microbiomes which in its genomic essence is the combined genetic material of the microorganisms inhabiting an environment, whether the environment be a particular body econiche (e.g., human intestinal contents) or a food manufacturing facility econiche (e.g., floor drain). To date, 16S rDNA sequencing, metagenomics and metatranscriptomics are the three basic sequencing strategies used in the taxonomic identification and characterization of food-related microbiomes. These sequencing strategies have used different NGS platforms for DNA and RNA sequence identification. Traditionally, 16S rDNA sequencing has played a key role in understanding the taxonomic composition of a food-related microbiome. Recently, metagenomic approaches have resulted in improved understanding of a microbiome by providing a species-level/strain-level characterization. Further, metatranscriptomic approaches have contributed to the functional characterization of the complex interactions between different microbial communities within a single microbiome. Many studies have highlighted the use of NGS techniques in investigating the microbiome of fermented foods. However, the utilization of NGS techniques in studying the microbiome of non-fermented foods are limited. This review provides a brief overview of the advances in DNA sequencing chemistries as the technology progressed from first, next and third generations and highlights how NGS provided a deeper understanding of food-related microbiomes with special focus on non-fermented foods.

## Introduction

It is well known that foodborne diseases cause considerable morbidity and mortality in humans particularly in immunocompromised individuals and in young children (Stein et al., 2007; Tauxe et al., 2010). Many foodborne diseases are caused by bacteria, viruses, and parasites (Scallan et al., 2011). Consequently, sporadic infections or outbreaks are reported regularly in many countries. In addition, increased globalization has resulted in the transmission of foodborne pathogens across international borders, severely impacting trade and food security (Frank et al., 2011b; Bernard et al., 2014). This has led national governments and international bodies to establish elaborate controls to improve food safety. Therefore, there is a burden on different food production systems to provide food safe for consumption.

Food, an indispensable part of everyday life, undergoes many processing steps before reaching the consumer. The total population of all microorganisms (microbiome), play important roles in any food matrix ranging from fermentation, contamination and spoilage. Deep taxonomic understanding of the microorganisms and their communities is required either to enhance desired food processes like fermentation or to mitigate detrimental occurrences like contamination and spoilage. Historically, conventional techniques including the classical Gram stain along with individual biochemical characteristics are used for the isolation, identification and characterization of bacteria from clinical, food or environmental origins. Even though considered as the “gold standard,” culture dependent techniques can only detect 0.1% of a complex community, such as that found in the human intestinal microbiota. Therefore, to extend the understanding of an ecological niche, such as food, techniques are needed to identify or characterize microorganisms and predict the functional interactions of different microbiological communities present in the sample. To this end, contemporary advances in multi-omic technologies have enabled microbial community profiling, monitoring population fluctuations in different microbial ecosystems and characterization of different microbial species in food matrices.

The rapid development of nucleic acid sequencing technologies over the past four decades has improved the capacity to characterize the microbiomes of complex matrices associated with food or environmental samples. The ubiquitous nature and specificity of nucleic acids make the molecule an ideal target for bacterial or microbiome characterization. Utilizing significant advancements in sequencing chemistries, DNA sequencing gradually evolved from low throughput DNA fragment sequencing to high throughput next generation (NGS) and third generation sequencing techniques (Loman and Pallen, 2015).

Traditionally, most NGS related food microbiome studies have focussed on fermented foods, such as cheese, kimchi and sausages (Patra et al., 2016; De Filippis et al., 2017). Different studies have not only enabled the characterization of the microbial composition of fermented foods but also identified the changes in microbial structure overtime, along with changes in the gene expression patterns related to different fermentation stages (Bokulich et al., 2012; Jung et al., 2013; Ahn et al., 2014; Lessard et al., 2014; Połka et al., 2014). Compared to fermented foods, studies to identify and characterize the microbiome of non-fermented foods are scarce. This review will present the basic principles of the currently available DNA sequencing techniques and how some of these strategies played a key role in understanding microbiomes associated with food, with special emphasis on non-fermented foods.

## Whole genome sequencing

Scientific advances in whole genome sequencing proceeded through three major technological revolutions: first *generation sequencing* (whole genome shotgun sequencing), *next generation sequencing* (NGS high throughput sequencing) and the *third generation of sequencing* (single molecule long read sequencing) (Loman and Pallen, 2015).

### First generation DNA sequencing (whole genome shotgun sequencing)

The first DNA sequencing strategy was the *Sanger Chain Termination Method*. Whole genome shotgun DNA sequencing is a capillary based, semi-automated version of the original Sanger strategy. Here, DNA is randomly fragmented, cloned into high copy number plasmid and transformed into *Escherichia coli*. The cloned region is amplified using flanking PCR primers. Each PCR cycle is stochastically terminated by the incorporation of a fluorescently labeled dideoxyribonucleotide (ddNTP), corresponding to the nucleotide identity at the terminal position. DNA fragments are then separated in a high resolution electrophoretic capillary containing polymer gel, and upon exit from the gel, the fluorescent label is excited by an argon laser and the emission spectrum is captured. Read lengths of approximately 1,000 base pairs were obtained with an accuracy of 99.99%. However, low throughput results together with high operational costs, restricted the application of this method.

### NGS high throughput sequencing

The advantages of NGS over Sanger sequencing can be summarized as follows (1) *in vitro* construction of the sequencing library; (2) *in vitro* clonal amplification of DNA fragments; (3) array based sequencing enabling DNA fragments to be multiplexed (4) solid phase immobilization of DNA. Based on the different methods used to immobilize DNA on a solid substrate, three technologies were commercialized; (a) high throughput pyrosequencing on beads, (b) sequencing by ligation on beads and **(c)** sequencing by synthesis on a glass substrate.

***(a) High throughput pyrosequencing on beads***. The first next-generation high throughput sequencer to be made commercially available was the 454 GS20 pyrosequencing platform (Roche) (Margulies, 2006). A DNA molecule is first sheared with enzymatic based digestion or sonication and ligated with oligonucleotide adapters. Each ligated fragment is then attached to a 28-μm bead, PCR amplified in an oil-water emulsion and pyro sequenced (Ronaghi et al., 1996). Amplicon bearing beads are then captured in a picolitre sized well and the immobilized DNA fragments are pyrosequenced. In each pyrosequencing cycle, the addition of an unlabelled nucleotide will result in the enzyme-mediated release of an inorganic pyrophosphate (PPi) molecule that is detected computationally. These iterative pyrogenic cycles generate a DNA sequence with a mean read length of 400 nucleotides. The main disadvantage of this technique is reading through homopolymeric sequences, where on occasion n nucleotides are read as n-1 nucleotides, making this technology prone to high error rates. *Myxococcus xanthus*, a soil inhabitant, was the first bacterium to be sequenced using this technology (Vos and Velicer, 2006). Subsequently this method was used in a survey of microbial populations from different environments *viz*. underground mine water, marine, fresh water, fish, corals terrestrial animals and mosquitoes (Dinsdale et al., 2008).
***(b) Sequencing by ligation on beads***. SOLiD technology was based on the Multiplex Polony Sequencing technology (Shendure et al., 2005). Adaptor flanking template DNA fragments were initially attached to 1-μm paramagnetic beads and PCR amplified in an oil-water emulsion. Beads with attached PCR amplicons were immobilized on a solid planar substrate and hybridized with a universal PCR primer complementary to the adaptor. Each sequencing cycle proceeds through the ligation of a fluorescently labeled DNA octamer to the universal primer revealing the positional identity of the nucleotide. Subsequent chemical cleavage leaves a pentamer on the DNA template. Progressive iteration of this process reveals the DNA sequence. Since this platform utilizes a two-base coding system, miscalls are more readily identified resulting in 99.94% accuracy.
***(c) Sequencing by synthesis on a glass solid phase surface***. The Illumina Genome Analyser (SOLEXA) was described in 2006 and 2008 (Fedurco et al., 2006; Turcatti et al., 2008). The DNA library preparation involves random fragmentation of template DNA and the ligation of oligonucleotide adaptors. The DNA amplification strategy involved is referred to as Bridge PCR (Adessi et al., 2000; Fedurco et al., 2006). Both forward and reverse primers, with complementarity to the adaptor, are attached to a glass surface by a flexible linker. The adaptor flanked DNA fragments are hybridized on to the forward and reverse primers attached to the glass surface. Bridge PCR then amplifies the DNA fragment using formamide based denaturation and *Bst* DNA polymerase, resulting in a “*cluster*” of clonal amplicons. Amplicons produced from a single DNA fragment will cluster in a single physical location on the array. Following cluster generation, the sequencing primer hybridizes to the universal sequence flanking the region of interest. Sequencing then proceeds in cycles with a modified DNA polymerase and four nucleotides. Nucleotides are labeled with a chemically cleavable fluorescent reporter group at the 3′-OH end thereby allowing only a single base incorporation in each cycle. Each cycle extends a single base followed by the chemical cleavage of the fluorescent reporter that will identify the incorporated nucleotide. Advanced chemistry designs have allowed paired end reads of 2 × 300 bp from each DNA fragment. The basic challenges of Illumina technology are signal decay and dephasing caused by incomplete fluorescent label cleavage or terminating moieties. Average raw error rates are of the order 1–1.5%.

### Third generation sequencing (single molecule long read sequencing-SMRT)

A recognized limitation of NGS technologies is the requirement for a PCR amplification step, which introduces a bias in read distribution ultimately affecting the coverage. Third generation sequencing technology was designed to address this limitation. Here single DNA molecules are directly sequenced thereby reducing low error rates by avoiding amplification associated bias, intensity averaging, phasing or synchronization problems.

The first commercially released long read methodology was single-molecule-real-time (SMRT®) technology (Pacific Biosciences) (Eid et al., 2009). In this case the library preparation step constructs a closed circular DNA molecule by ligating an adaptor molecule to both ends of the target DNA molecule to be sequenced. The circular DNA molecule is then loaded into a SMRT® cell containing 150,000 zeptolitre wells. Each well has a single immobilized DNA polymerase at its base. DNA polymerase then binds to the hairpin adaptors on the circular target DNA molecule and initiates replication. Four fluorescently labeled nucleotides are then introduced into the reaction wells. As each base is enzymatically incorporated, a light pulse is produced that identifies the base and analyzed iteratively to generate the DNA sequence (Rhoads and Au, 2015). The main advantage of the SMRT® sequencing is the read length obtained. The original C1 generation sequencer produced a read length of about 1,500 bp. More recent C4 chemistry protocols provided for an average read length of 10-kbp. The typical throughput of a PacBio RS II system is 0.5–1 billion bases per SMRT® cell. However, the platform has significantly higher error rates (approximately 11- to 15%).

HeliScope (Braslavsky et al., 2003) is another example of single DNA molecule sequencing. In that case single DNA molecules are sequenced by synthesis using a highly sensitive fluorescence detection system. A DNA library is prepared by random DNA fragmentation followed by poly A tailing. The poly A tail is then hybridized to surface tethered poly T oligomers. This yields an array of primer annealed single molecule DNA templates. To this primer, DNA polymerase adds a single nucleotide resulting in a template-dependent extension. Each nucleotide has a fluorophore attached and these are introduced one nucleotide at a time. The recorded image is analyzed to identify the nucleotide being incorporated into the growing strand. The cycle is then repeated with a new species of nucleotide.

MinION (Oxford Nanopore Technology) was released in 2014 through the MinION Access Programme (MAP). Here, electrophoresis is used to move the DNA/RNA molecule through a nanopore. This system involves the use of electrolytic solutions and the application of a constant electric field. As the nucleic acid passes through the nanopore, the change in the current pattern and magnitude is measured. During the library preparation step, double stranded DNA is sheared using a Covaris g-TUBE and fragmented DNA is repaired using a PreCR step. Blunt ended DNA molecules are then created using an end repair step before a poly A tail is added to the 3′-OH end. Two adaptors are then added to the DNA, a Y adapter (so called, due to its shape) and a hair pin adaptor. A motor protein unzips the double stranded DNA at the Y adapter and feeds the DNA as a single strand through the nanopore. Base calling is then performed and a read length of a few hundred thousand base pairs is achieved with an accuracy ranging from 65 to 88%. If information from only one strand is used, base calling is 1-dimensional (1D), otherwise it is 2-dimensional (2D) system (Lu et al., 2016). Due to the small size of the instrument, low cost and the real-time nature of this platform, the MinION platform is attracting interest in the genomics community particularly for pathogen surveillance and diagnostics (Judge et al., 2015; Quick et al., 2015).

## Sequencing techniques applied to characterize food-related microbiomes

### 16S rDNA sequencing

This is one of the most important culture independent methods used for conventional microbiome analysis. Most bacteria contain 16S rDNA gene which is made up of nine hypervariable regions flanked by conserved sequences (Neefs et al., 1993). This offers a unique opportunity to design generic PCR primers to amplify and sequence these hypervariable loci to identify the corresponding bacterial taxonomy of the species associated with the food matrix. Similarly, the 18S rDNA gene can be used to identify fungi. Based on the nucleotide sequence similarity, these sequences are clustered into Operational Taxonomic Units (OTU). OTUs are then compared against databases to identify the microorganisms present in the microbiome. The first attempt to characterize a microbiome utilizing this approach was the identification of microbial population from Sargasso sea picoplankton (Giovannoni et al., 1990).

Traditional Sanger sequencing allows only a smaller proportion of amplicons to be sequenced. This results in less abundant members of the microbiome population being missed, thus compromising the comprehensive description of the microbial community. The subsequent inclusion of NGS platforms in 16S rDNA sequencing increased the capacity for a more thorough identification of the bacterial members of the community by several orders of magnitude and at a much lower cost. Since only a short amplicon was sequenced, much higher coverage per sample was obtained (Claesson et al., 2009). In addition, pyrosequencing allowed individual samples to be indexed and facilitated multiplexing during each instrument run (Hamady et al., 2008). This latter step provided a breakthrough in the way environmental prokaryotes could be analyzed. Since then 16S rDNA sequencing has become one of the most popular techniques to identify the microbiome members associated with food matrices. One of the main advantages of using the 16S rDNA sequencing approach is the availability of many bioinformatic tools designed for sequencing data analysis which are free and easy to operate. Commonly used software to analyze 16S rDNA data from food/environmental samples include QIIME (Quantitative Insights Into Microbial Ecology) (Caporaso et al., 2010), mothur (Schloss et al., 2009), and USEARCH (ultra-fast sequence analysis) (Edgar, 2010).

Due to shorter reads obtained from NGS protocols, especially from Illumina platforms, bacterial classification using 16S rDNA sequencing often cannot be identified beyond the genus level (Claesson et al., 2010). Furthermore, 16S rDNA sequencing was found to underestimate the contribution of Gram negative bacteria when compared to bacterial counts observed using transmission electron microscopy and Gram staining (Hugon et al., 2013). A further challenge to this approach is related to the choice of nine hypervariable regions (V1–V9) contained within the 16S rDNA gene. The selection of the hypervariable region for 16S rDNA sequencing has not been generally dependent on the sample environment but rather on published or in-house designed protocols. Many authors have favored different hypervariable regions, such as V1/V2/V4 (Sundquist et al., 2007), V2/V3/V4 (Liu et al., 2008), V2/V4 (Wang et al., 2007), and V2/V3 (Chakravorty et al., 2007). A systematic survey of the efficiency of different hypervariable regions identified that PCR primer pairs targeting V4/V5 was best to identify the microbiome with reduced amplification bias compared to the standard V3/V4 (Claesson et al., 2010). PCR amplicon fragments as short as 82 bp targeting the 16S rDNA V5 variable region have proved to be of sufficient length for bacterial classification at the phylum level (Lazarevic et al., 2009), and a longer amplicon fragment of 100 bp combined with proper primer design and downstream analysis was capable of displaying the same clustering information as longer 16S rDNA sequence (Liu et al., 2007). Even with longer variable regions, pyrosequencing and higher coverage, the amplification of different polymorphic regions resulted in a bias in assessing the microbiome. Moreover, the focus on one marker gene, neglecting other genomic biomarkers makes this technique unsuitable for isolate-specific identification. Nevertheless, considering the low cost per sample and the requirement of low input template DNA concentrations, 16S rDNA sequencing remains one of the most popular high-throughput sequencing methods.

### Metagenomic and metatranscriptomic sequencing

Metagenomics refers to the application of high throughput techniques to sequence the entire DNA (or RNA) content found in a sample, independent of its origin. Template DNA contained in a sample of interest is subject to sequencing directly, without any prior marker gene amplification step. Metagenomic data not only provides an in-depth taxonomic identification of the microbiome but can also simultaneously compare the relative abundance of all organisms present in the microbiome. Substantial amounts of sequencing data generated using a metagenomic approach is then queried against databases, such as k-mer (Compeau et al., 2011) and SILVA (Quast et al., 2013) to determine the taxonomic composition of microorganisms within the sample. The main advantage of metagenomic approaches over 16S rDNA sequencing is the ability to characterize bacteria present in the microbiome to their species/strain level. In addition, metagenomics also provides comprehensive information on the entire repertoire of genes, structure and organization of the genomes, microbial community structure and evolutionary relationships present in the sample. Consequently, this approach has significant advantages over the 16S rDNA marker gene approach.

The main challenge of the metagenomic approach is the amount of sequence data generated. This procedure is expensive compared to 16S rDNA-based strategies. Moreover, data analysis requires high-end bioinformatics requiring a long term financial investment, which is possible only in specialized laboratories. The lack of specially designed reference databases also makes the use of this technology challenging when attempting to extract biological information on a routine basis.

Another concern is that metagenomics approaches cannot distinguish viable microbial populations within a microbiome (Ercolini, 2013). Treatment of such samples with propidium monoazide (PMA) has been demonstrated to be a useful approach to distinguish the viable members of a microbiome (Wagner et al., 2008; Erkus et al., 2016). PMA selectively binds to free DNA present in the matrix and as this compound cannot penetrate a cell with an intact cell membrane, it targets the substrate from dead bacteria and other cells. Treating a food matrix sample with PMA prior to DNA extraction will enable the binding of PMA to the free DNA present in the sample and this step will inhibit subsequent sequencing. DNA from viable cells are unaffected and are thus available to be subsequently sequenced.

Alternatively, RNA can also be used as a template to distinguish the viable population within the microbiome. Sequencing total RNA purified from a sample is the basic principle underpinning metatranscriptomic analysis. Apart from distinguishing the viable members within the population contained in a microbiome, this technique is invaluable for providing a functional characterization of the different bacterial members of the microbiome. In a complex microbiological sample, such as food, different microbiological communities interact with each other, either to degrade, spoil or ferment the organic constituents of the matrix. Sequencing RNA purified from these samples would provide a basic description of how the communities interact with each other.

## Application of NGS techniques in selected microbiome studies

Determining the optimal sequencing approach useful for the study of different food matrices depends upon the complexity of the sample to be analyzed and the depth of bacterial taxonomic information required. An initial 16S rDNA sequencing based profile would provide a broad overview of the microbial composition within a food sample. Nonetheless, this technique lacks the necessary resolution required to provide species-level/strain-level identification. Further, it will not provide an assessment of the functional capability of these organisms, contained within the sample. Therefore, for in-depth species-level or for strain level identification or detailed functional characterization of the different members in the microbiome, metagenomics and metatranscriptomics would be useful.

### Examples of different sequencing approaches used to characterize food microbiomes

For the purpose of this review, a literature search was performed on the current NCBI PubMed database (https://www.ncbi.nlm.nih.gov/pubmed) using a group of seven phrases as follows: (1) 16S rDNA sequencing food microbiome, (2) 16S rRNA sequencing food microbiome (3) 16S metagenetics, (4) metagenomic food sequencing, (5) metagenomic food, (6) metatranscriptomic food and (7) metatranscriptomic food sequencing.

**Figure 1:**
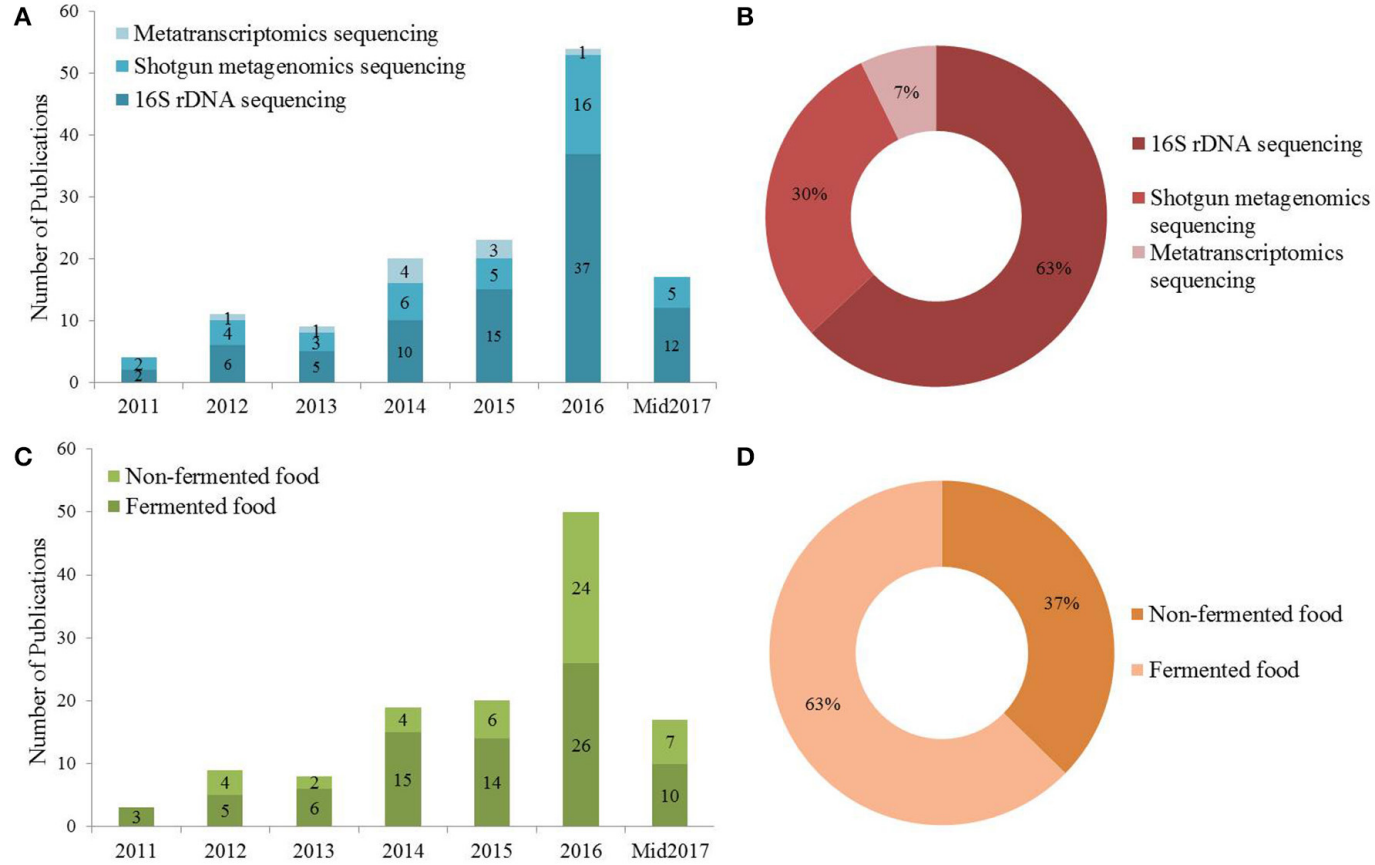
**(A)** The total number of publications utilizing NGS technology investigating the microbiome associated with fermented and non-fermented food between 2011 and June 2017. **(B)** The proportion of different sequencing strategies used in the publications mentioned in **(A)**. **(C)** The number of publications utilizing NGS strategies to investigate fermented and non-fermented food. **(D)** The percentage of publications utilizing NGS approaches in investigating fermented and non-fermented foods.

Between 2011 and June 2017, a total of 126 papers were published describing the characterization of various food microbiomes (Figure 1A). Of all the publications using different NGS techniques in the analysis of food microbiome more than half (63%) used 16S rDNA sequencing, suggesting that this strategy was widely applied for the analysis of food/food production related studies (Figure 1B). NGS-based methods were more often applied to characterize fermented (63%) type food matrices compared to non-fermented foods (Figures 1C,D). Since 2012, metagenomic and metatranscriptomic approaches were more often used for food microbiome characterizations and the numbers of studies using these latter approaches gradually increased (Figure 1A). The utility of high throughput sequencing approaches in fermented foods was extensively reviewed previously (Patra et al., 2016; De Filippis et al., 2017). To provide a contrast to these data, this review focuses on the compositional analysis of microbiomes associated with non-fermented foods.

### Application of NGS-based strategies to raw materials and production environment surveillance

Hygiene is an essential step in the *farm-to-fork* continuum and all production/processing lines are designed to maintain sanitary standards at all stages of food manufacture. Improvements in the maintenance of sanitary standards have enhanced food safety by reducing the number of pathogenic bacteria colonizing these environments and ultimately cross contaminating the final food product. The quality of the final food product not only depends on the hygiene of the processing environment but also on the quality of the raw materials used. For example, a 16S rDNA sequencing-based approach reported that milk with higher somatic cell counts was associated with a higher abundance of certain bacterial taxa including *Acinetobacter, Enterobacteriaceae, Corynebacterium*, and *Strepococcus* species (Rodrigues et al., 2017), along with *Thermoanaerobacterium*, a genus identified for the first time in the core microbiome of a milk sample. The presence of spoilage organisms (such as *Acinetobacter, Thermoanaerobacterium* among others) along with pathogenic bacteria (including *Enterobacteriaceae, Corynebacterium* species, and *Strepococcus*) in the raw material can potentially contaminate the resident microbiome of bulk milk tank in which the raw material is stored representing a threat to the entire downstream production line. The influence of the ecology of processing environments on food quality was shown in a more elaborate study involving bulk milk tankers (Kable et al., 2016). Using a 16S rDNA sequencing-based approach, it was shown that the microbiome of milk containing silos was influenced by that of the bulk milk tankers feeding them, and that distinct bacterial communities were evolved within different milk silos. Furthermore, the study showed that the composition of the milk microbiome varied seasonally, as higher bacterial diversity was associated with the spring season. These observations, however, were not limited to dairy facilities. In cheese production sites, the *in house* environmental flora dominated the cheese microbiome (Bokulich and Mills, 2013). This demonstrated that the presence of spoilage microorganisms in the immediate environment of the processing line increase the chances of subsequent spoilage of the final product. Identification of spoilage bacteria, such as *Pseudomonas, Acinetobacter* and *Psychrobacter* species were found along with the core microbiota, indicating the potential of these bacteria to contaminate the processing line (Stellato et al., 2015). Clearly, the hygienic status of the processing facility is a crucial factor that can influence the microbiome of the food matrix. A comprehensive list of publications utilizing NGS approaches in the identification of microbiomes associated with food production chains is given in Table 1. In an effort to gain a better understanding of the microbiome of a powder infant formula (PIF) processing facility, 16S rDNA sequencing approach was used to identify the microbiome of the designated low, medium and high care areas (Anvarian et al., 2016). The medium care area had the highest bacterial diversity, possibly due to the moisture associated with these locations. These studies provide a novel insight into the dynamics of the microbiome and demonstrate how storage, processing or temporal fluctuations in the production environment may influence the microbiological quality and safety of food products.

**Table 1:** A comprehensive list of publications using next generations sequencing approaches to study the environmental microbiome along the food production chain.

**Research target**	**Country**	**Methods**	**Sequencing platform**	**Conclusions**	**References**
Artisan cheese factory and cheese samples	United States	16S rDNA sequencing (V4); qPCR	Illumina MiSeq	Facility-specific “house” microbiota play an important role in shaping site-specific characteristics in products	Bokulich and Mills, 2013
Wine factory equipment surface	United States	16S rDNA sequencing (V4)	Illumina MiSeq	Winery surface microbiomes have no obvious link with spoilage microbes in wine under normal operating conditions	Bokulich et al., 2013
Carcass, processing environment and beefsteaks	Italy	16S rDNA sequencing (V1-V3)	Roche 454 GS Junior	4°C aerobic storage led to dramatic decrease in beef microbial complexity; spoilage-associated bacteria originated from carcasses and carried through the production chain to the products	De Filippis et al., 2013
Brewery plant environment and beer product	United States	16S rDNA sequencing (V4, for bacteria); Fungal internal transcribed spacer (1 loci, for fungi); T-RFLP; Droplet digital PCR)	Illumina MiSeq	Most microbes found in the brewery environment originated from raw ingredients; beer-spoilage and hop-resistance genes were found throughout the brewery, but little beer spoilage occurred	Bokulich et al., 2015
Sausage processing environment and product	Finland	16S rDNA sequencing (V1-V3)	Roche 454 Titanium FLX	Abundant mesophilic psychrotrophs were prevalent throughout sausage production chain microbiomes, and with different characteristic patterns of contamination for different genera	Hultman et al., 2015
Ready-to-eat meal plant environment and product	Not mentioned	16S rDNA sequencing (V1-V3)	Roche 454 GS Junior	*L. gelidum* was identified to be dominant in ready-to-eat meal samples at the end of shelf-life, its spoilage characteristic and ability of growing under cold storage should raise industries' concern	Pothakos et al., 2015
Cheese factory environment and cheese product	Italy	16S rDNA sequencing (V1-V3, for bacteria); 26S rDNA sequencing (D1-D2, for fungi)	Roche 454 GS Junior	Coexistence of lactic acid bacteria and possible spoilage-associated bacteria was found in core microbiota of cheese factory environment and cheese samples	Stellato et al., 2015
Powdered Infant Formula plant environment	Ireland	16S rDNA sequencing (V3-V4); Flow cytometry	Illumina MiSeq	Bacteria present in low, medium and high care area of a powdered infant formula plant environment were mostly associated with soil, water, and humans, respectively	Anvarian et al., 2016
Environment samples alone beef production chain	United States	Shotgun metagenomics sequencing	Illumina HiSeq 2000	No antimicrobial resistant determinants (ARD) were identified in final beef products, indicating slaughter interventions may reduce ARD transmission risk	Noyes et al., 2016
Dairy farm agroecosystems	United States	Shotgun metagenomics sequencing	Ion Torrent Personal Genome Machine	The most abundant antimicrobial resistant genes in dairy agroecosystems were grouped under multidrug transporters	Pitta et al., 2016
Butchery meat and environment samples	Italy	16S rDNA sequencing (V1-V3)	Roche 454 GS Junior platform	The type of retail (large- or small-scale distribution) had no apparent effect on initial fresh meat contamination	Stellato et al., 2016
Environment samples along beef production chain	United States	Shotgun metagenomics sequencing	Illumina HiSeq 2000	Usage of standard antimicrobial interventions in beef processing system significantly reduced the diversity of remaining microbiomes	Yang et al., 2016

### Monitoring the surface microbiome of ready-to-eat food product(s)

Vegetables are known to be vehicles of pathogenic microorganisms and in several cases and have led to outbreaks of foodborne illness (Buchholz et al., 2011; Frank et al., 2011a). Farm waste water used for irrigation contains high numbers of coliform bacteria and is one of the factors responsible for the surface contamination of vegetables (Van Dyk et al., 2016). Phyllosphere, the part of plant surface located above ground, is colonized by several microorganisms (Lindow and Brandl, 2003). Recent studies using NGS-based methods have identified how the phyllosphere microbiome can be affected by season, irrigation, soil type and other parameters. The most direct correlation between the composition of a plant microbiome with temporal fluctuations was shown in the case of the Romaine lettuce phyllosphere microbiome (Williams et al., 2013). Firmicutes dominated the microbiome in June whilst proteobacteria dominated in the period August-October. In addition to temporal fluctuations, different plant irrigation methods also influence the compositions of plant microbiome. For example, in a study conducted on tomato plants, when irrigated with ground water, the microbiome contained mainly Proteobacteria while surface water gave rise to the plant microbiome containing Firmicutes, Actinobacteria, and Verrocomicrobia (Telias et al., 2011).

Soil is another factor that influences vegetable and fruit microbiomes. A study using 16S rDNA sequencing reported that the microbiomes of leaves, flowers and fruits shared a greater proportion of taxa with the soil microbiome in which the plants were grown (Zarraonaindia et al., 2015). Therefore, irrigation water containing pathogenic microorganisms may contaminate the soil resulting in cross-contaminating vegetables or fruits (Van Dyk et al., 2016), which led to outbreaks (Buchholz et al., 2011). Each plant product may also harbor bacterial communities that are unique (Leff and Fierer, 2013). Sprouts, spinach, lettuce, tomato, pepper and strawberries exhibited a high abundance of Enterobacteriaceae, whilst other fruits like apples, peaches, grapes and mushrooms were found to be dominated by Actinobacteria, Bacteriodetes, Firmicutes and Proteobacteria. Since all these food product types are consumed with minimal processing, individuals can be exposed to these bacterial phyla. A comprehensive list of publications utilizing NGS approaches in the identification of microbiomes associated with raw and ready to eat food products is given in Tables 2, 3, respectively.

**Table 2:** A comprehensive list of publications using next generations sequencing approaches used in characterizing the microbiome of raw food products.

**Research target**	**Country**	**Methods**	**Sequencing platform**	**Conclusions**	**References**
Broiler filet strips	Finland	16S rDNA sequencing (V1-V3); T-RFLP	Roche 454 GS FLX	Marination process led to increased lactic acid bacteria in broiler meat microbiome, resulting in enhanced CO_2_ production and acidification	Nieminen et al., 2012b
Broiler filet strips	Finland	16S rDNA sequencing (V1-V3); Shotgun metagenomics sequencing	Roche 454 GS FLX; Roche 454 GS FLX;	Marination altered broiler fillet strips' microbial community by favoring the spoilage associated bacteria *L. gasicomitatum*	Nieminen et al., 2012a
Spoiled retail foodstuffs	Belgium	16S rDNA sequencing (V1-V3)	Roche 454 GS Junior	Characterization of psychrotrophic lactic acid bacteria that cause unexpected food spoilage cases in Belgian retail food	Pothakos et al., 2014
Store bought meat	United States	Shotgun metagenomics sequencing	Illumina Miseq	Primary characterization of viruses commonly found in US store-bought meats	Zhang et al., 2014
Beef burger	Italy	16S rRNA sequencing (V1-V3); PCR-DGGE	Roche 454 GS Junior	Nisin-based antimicrobial packaging reduced the abundance of microbes that produce compounds of specific metabolic pathways related to spoilage	Ferrocino et al., 2016
Raw pork sausage	France	16S rDNA sequencing (V1-V3); qPCR	Roche 454 GS FLX++ Titanium	Salt reduction, particularly when combined with CO_2_-enriched packaging, resulted in faster spoilage of raw sausages by lowering the overall bacterial diversity	Fougy et al., 2016
Raw milk	Finland	16S rDNA sequencing (V1-V2);	Illumina MiSeq	Bacterial diversity is better preserved in bovine raw milk by additional flushing with N_2_ gas compared to cold storage at 6°C alone	Gschwendtner et al., 2016
Raw milk	United States	16S rDNA sequencing (V4); qPCR	Illumina MiSeq	raw milk microbial community structure can be influenced during low-temperature, short-term storage	Kable et al., 2016
porcine musculature	Austria	16S rDNA sequencing (V1-V2); qPCR	Roche 454 GS-FLX Titanium	Pork sample microbiota was dominated by psychrophilic spoilers; *E. coli* was present in all pork samples and can be used as marker species in pork contamination cases	Mann et al., 2016
Raw milk	Australia	16S rDNA sequencing (V5-V8)	Roche 454	Spoilage bacteria growth was delayed by at least 7 days in CO_2_ treated raw milk sample	Lo et al., 2016
Bulk tank milk	United States	16S rDNA sequencing (V4); qPCR; Flow cytometry	Illumina MiSeq	Spoilage and spore-forming bacteria were ubiquitous in all dairy farms	Rodrigues et al., 2017
Common carp filets	China	16S rDNA sequencing (V3-V4)	Illumina HiSeq 2500	Use of cinnamon essential oil extended vacuum-packaged common carp fillets shelf-life by approximately 2 days based on sensory and other analysis, but showed no significant differences in dominant microbiota composition compared with non-treated samples at the end of shelf-life	Zhang et al., 2016

**Table 3:** A comprehensive list of publications using next generations sequencing approaches in characterizing the microbiome of ready to eat food.

**Research target**	**Country**	**Methods**	**Sequencing platform**	**Conclusions**	**References**
Bagged leaf vegetables	United States	16s rDNA sequencing;	Roche 454 GS-FLX Titanium	No significant differences found on microbial compositions between organic and conventionally grown, surface-sterilized and non-sterilized leaf vegetables	Jackson et al., 2013
Store-bought fruits and vegetables	United States	16S rDNA sequencing	Roche 454	Microbial communities of certain type product are more similar than different types, but Significant difference identified between conventional and organic product within the same type	Leff and Fierer, 2013
Field grown lettuce	United States	16S rDNA sequencing (V5-V9); qPCR	Roche 454 GS-FLX Titanium	Lettuce phyllosphere microbiome are affected by seasonal, irrigation, and biological factors	Williams et al., 2013
Carrots	United Kingdom	Metatranscriptomics qPCR	Illumina MiSeq	Carrot yellow leaf virus are strongly associated with carrot internal necrosis	Adams et al., 2014
Basil leaves	Belgium	16S rRNA sequencing (V1-V3); PCR-DGGE	Roche 454 GS-FLX Titanium	Spoilage of commercially grown basil leaves was caused by tissue injuries and visual defects rather than by specific bacterial growth	Ceuppens et al., 2015
Cilantro	United States	16S rRNA sequencing (V1-V3); Shotgun metagenomics sequencing (with pre-enrichment)	Illumina MiSeq; Illumina MiSeq	A 24 h non-selective enrichment identified *Salmonella* spiked in cilantro	Jarvis et al., 2015
Bagged spinach	United States	Shotgun metagenomics sequencing (with pre-enrichment)	Illumina MiSeq	Eight h pre-enrichment and sequencing depth identified' spiked Shiga toxin-producing *E. coli* as low as 100 CFU/100 g in bagged spinach	Leonard et al., 2015
field-grown and retail lettuce	United States	Shotgun metagenomics sequencing; Metatranscriptomics	Illumina HiSeq 2500; Illumina HiSeq 2500	Virome of iceberg lettuce from fields and produce distribution center were dominated by plant pathogenic viruses but human and animal viruses were also identified	Aw et al., 2016
Oregano	United States	Shotgun metagenomics sequencing (with pre-enrichment)	Illumina MiSeq	Addition of corn oil during pre-enrichment of oregano samples led to increased overall abundance of Gram negative microorganism and a ≥50% recovery rate of *Salmonella*	Beaubrun et al., 2016
Bagged spinach	United States	Shotgun metagenomics sequencing (with pre-enrichment)	Illumina MiSeq	Shotgun metagenomics sequencing identified Shiga toxin-producing *Escherichia coli* (STEC) spiked into fresh bagged spinach	Leonard et al., 2016
Cheese	Ireland	16S rDNA sequencing (V4-V5); Shotgun metagenomics sequencing; qPCR	Roche 454 GS-FLX; Illumina HiSeq 2000	Carotenoid-producing bacteria, genus *Thermus*, is linked with pink discoloration defect in cheese	Quigley et al., 2016

All the above studies provide insights into the role that NGS-based strategies played in uncovering the dynamic changes in the microbiome. Careful analysis of NGS data can be used to facilitate the development of safer production processes thereby reducing risk for the consumer.

### Monitoring microbiomes associated with food storage conditions

Control measures like refrigeration, modified atmospheric packaging (MAP), nisin treatment and others are often used to extend the shelf life of many perishable food products. The microbiome undergoes considerable compositional change when food products are stored under defined conditions. It is possible that these fluctuations in the microbiome may finally affect the quality of the food product. NGS techniques have been increasingly applied to study how these variations contribute to improved shelf life. The most common response noted in refrigerated food products was a reduction in bacterial diversity associated with the microbiome. A 16S rDNA sequencing approach noted this reduction in bacterial diversity in refrigerated spinach (Lopez-Velasco et al., 2011). Long-term storage of spinach at low temperatures selected for *Pseudomonas* species and *Enterobacteriaceae* family members. Similarly in beef steaks, reduction in bacterial diversity correlated with the increase in the abundance of spoilage agents like *Pseudomonas* species and *Brochothrix thermosphacta* (De Filippis et al., 2013). These bacterial species contaminated the raw meat during animal slaughter and proliferated during refrigerated storage. Since bacterial diversity is reduced during refrigeration, the competition for nutrients is similarly reduced. This development provides an opportunity for spoilage organisms to overgrow resulting in deterioration of the food product following long-term refrigerated storage.

Marination is another traditional treatment method frequently used during food production process. It is a process of soaking foods in a seasoned, often acidic, liquid before cooking. The derivation of the word refers to the use of brine or a water solution containing a significant amount of salt. The most common examples are used for curing, preserving, and developing flavor in foods, such as that used in the pickling process. NGS-based approaches have been used to investigate the microbiome diversity and structure alterations during marination, and to determine whether, such process can extend product shelf life. Sometimes contrary to its intended use, marination increased the speed of spoilage. In a typical example, the poultry product was rapidly spoiled when marinated with acetic acid and subsequently packaged in a modified atmospheric environment. Investigation on the bacterial composition contributing to spoilage using 16S rDNA sequencing indicated a heterofermentative lactic acid bacteria *Leuconostoc gasicomitatum* being detected as the spoilage organism. It was also noted that the process of marinating meat diminished the proportion of *Brochothrix thermosphacta, Clostridium* species, and *Enterobacteriaceae* in the sample, while increasing the proportion of spoilage associated *Leuconostoc gasicomitatum* (Nieminen et al., 2012a) which contributed to faster spoilage. The same study used metagenomics to identify *Vagococcus* species and *Vibrio* species both of which are predominating in un-marinated meat, a hitherto unobserved phenomenon.

Extended shelf life can also be achieved through the addition of the bacteriocin nisin, a polycyclic antibacterial peptide secreted by *Lactococcus lactis* which is applied to suppress the growth of spoilage and pathogenic microorganisms. 16S rDNA analysis confirmed that nisin treatment showed remarkable reduction in nisin susceptible taxa including *Kocuria rhizophila, Staphylococcus xylosus, Leuconostoc carnosum*, and *Carnobaterium divergens* (Ferrocino et al., 2016). The extension in shelf life could also be associated with the suppression of bacteria including *C. divergens*, a spoilage bacterium associated with refrigerated fish and meat products (Borch et al., 1996).

Addition of NaCl to meat is known to improve texture, flavor and taste whilst also improving shelf life by reducing water activity. In one study using high salt concentration along with low temperature and CO_2_ enriched packing to improve the shelf life of sausage meat, the reduction of salt concentration was not surprisingly associated with faster spoilage in products. The 16S rDNA sequencing-based investigation revealed that reduction in salt concentration led to an overall reduction in bacterial diversity which caused faster spoilage. Improvements in sausage meat processing with higher salt concentrations combined with vacuum packaging increased the abundance of a subpopulation consisting of *Enterobacteriaceae, Enterococcaceae* and *Leuconostocaceae* families, which also helped with delayed spoilage (Fougy et al., 2016).

The selected studies cited above demonstrate the potential of NGS approaches and facilitated a better understanding of bacterial food spoilage. These sequencing based investigations not only identified the spoilage agent in many cases but also showed how different bacterial communities interacted with each other to counteract spoilage.

### The status of metagenomic and metatranscriptomic approaches in understanding food microbiome

Metagenomics/metatranscriptomics is also a valuable tool to understand how bacterial communities interact with each other in fermented foods. The first metagenomic study used a 454 GS FLX titanium platform to describe the microbiome of a ferment food, kimchi, with the predominant microbial population identified as *Leuconostoc mesenteriodes* and *Lactobacillus sakei* (Jung et al., 2011). Interestingly, a substantial proportion of bacteriophage DNA was also detected. Bacteriophage DNA were further identified in large proportions during the fermentation of shrimp, kimchi, sourdough and sauerkraut (Park et al., 2011), suggesting an important role for phages in the fermentative process. Even though the analysis of the *Lactobacillus sanfranciscensis* genome suggested phages could affect fermentation (Vogel et al., 2011), no role could be assigned for them during the fermentation of sourdough (Foschino et al., 2005). This simultaneous detection of phage- and bacterial-DNA would not have been possible by conventional 16S rDNA sequencing approaches, and was one of the main advantages of metagenomics over the former.

Metatranscriptomics also played a pivotal role in understanding the process of fermentation, especially the ripening of cheese. Camembert-type cheese ripening is driven by fungal microflora including *Geotrichum candidum* and *Penicillium camemberti*. Functional gene expression studies using metatranscriptomics identified that genes associated with metabolic pathways/cell growth/stress responses were differentially expressed during cheese ripening and that changes in expression patterns occurred over the first 2 weeks of the ripening period (Lessard et al., 2014). Metagenomics identified that genes responsible for amino acid catabolism were involved in flavor production. A combined effort utilizing metagenomics, metatranscriptomics and biochemical analysis aided the understanding of the process involved in surface ripened cheese (Dugat-Bony et al., 2015). In that case, metatranscriptomic data facilitated the characterization of the interactions between the dominant microbial species like *Lactococcus lactis, Debaryomyces hansenii, Geotrichum candidum, Kluyveromyces lactis*, and *Corynebacterium casei* during degradation of the dairy matrix. *Lactococcus lactis* produced excessive amounts of lactate from lactose and *Debaryomyces hansenii*/*Geotrichum candidum* up-regulated their lactate dehydrogenase genes, showing that lactate was metabolized by the latter. Genes associated with amino acid catabolism were also involved in the flavor production studies of Reblochon-type cheese, identifying the differences in amino acid catabolism between two yeast species, *Debaryomyces hansenii* and *Geotrichum candidum*, during cheese fermentation. The former took over 30 days to add its flavor constituents while the latter organism, added its flavor constituents during the first phase of ripening. An increase in ripening temperature promoted the expression of many genes including those involved in proteolysis, lipolysis and amino acid catabolism, consistent with the metabolomics profile and volatile organic compounds detected in cheese (De Filippis et al., 2016).

All these insights above showed that metagenomic/metatranscriptomic analysis provided an in-depth analysis of how microbial communities interacted with each other during fermentation. However, reports on the usage of these techniques in non-fermentative foods are rare, and metagenomics/metatranscriptomics approaches have not been utilized to their full potential in investigating food microbiomes of non-fermentative foods. Considering the limitations of 16S rDNA sequencing approaches, metagenomics/metatranscriptomics could be increasingly used in future to extend understanding of the functional microbiome of non-fermented food.

### The utility of NGS approaches in identification and characterization of specific bacteria from a microbiome

Many bacteria are non-culturable, either because they are unknown or they are known but not recoverable in the laboratory conditions. Genomic approaches have played a major role in understanding such non-culturable bacteria, and in some cases, have led to development of new media that can be subsequently used for their cultivation. A classic example is the case of *Tropheryma whipplei*, an organism that can only be grown within a human fibroblast cell line. Genome data from this organism was used to identify specific metabolic deficiencies, providing support to design an axenic growth medium which was in turn used to culture this bacterium (Renesto et al., 2003). A similar example is pink discoloration of cheese. Pink discoloration is a defect affecting cheese leading to significant loss of revenue to the dairy industry. Despite decades of research, the cause of pink discoloration remained elusive. Combining 16S rDNA shot gun metagenomics and quantitative PCR (qPCR), the spoilage bacterium associated with pink discolored cheese was identified as *Thermus thermophilus* (Quigley et al., 2016). The carotenoids produced by the bacterium were responsible for pink discoloration. Subsequent, successful culture of the bacterium from pink colored cheese confirmed the diagnosis.

NGS approaches can be utilized in the identification and characterization of specific pathogenic or spoilage bacteria from food or food production microbiomes. Some examples include the identification of *Leuconostoc gasicomitatum* as the spoilage agent in marinated poultry (Nieminen et al., 2012a), along with *Pseudomonas* species and *Brochothrix thermosphacta* in refrigerated beefsteaks (De Filippis et al., 2013). Unfortunately, 16S rDNA sequencing cannot provide any further characterization of the organism other than identification. Since whole genomes are sequenced, data from metagenomic approaches can be used to characterize any specific microorganism within a microbiome. This is particularly helpful when the organism is non-culturable. One of the earliest examples, using metagenomic approaches, was the construction of the complete genome of an non-culturable α-propteobacteria *Candidatus Liberibacter asiaticus*, the causative agent of Citrus huanglongbing (Duan et al., 2009). Genes associated with motility, transport and virulence were identified in this genome. Similar metagenomics approaches were used in another study to confirm that carrot yellow leaf virus was responsible for carrot internal necrosis (Adams et al., 2014). The most recent example is the determination of the draft genome of *Listeria monocytogenes* from an ice-cream microbiome using metagenomics (Ottesen et al., 2016).

Since the sequence data obtained from a metagenomics approach are detailed, these data could be used to characterize organisms other than bacteria in the microbiome. Metagenomics approaches were used to study the virome associated with store-derived beef, pork and chicken identified a novel bovine polyomavirus in beef and a novel gyrovirus species in chicken (Zhang et al., 2014). These reports highlight the utility of NGS approaches in the deep taxonomic characterization of the microbiome.

### Monitoring environmental spread of antimicrobial resistance genes

Dissemination of antibiotic resistance in human pathogens is a matter of global concern. Antibiotics are used in food-producing animals and aquaculture as therapeutic agents, for prophylactics and in some jurisdictions as growth promoters (Phillips, 2004). Although the growth promoting effects of antibiotics on livestock was known for years, there were no experimental data substantiating this claim. NGS played an important role in proving the growth promoting effect of penicillin in broiler chickens (Singh et al., 2013). 16S rDNA sequencing demonstrated an increased proportion of phylum Firmicutes and decreased proportion of phylum Bacteriodetes in the gut microbiota of chicken post penicillin treatment. A similar shift in gut microbiota was noted in obese- compared to lean-individuals, suggesting that penicillin treatment had an indirect growth promotion effect. Metagenomics based assays identified the origin and the distribution pattern of antibiotic resistance encoding genes in a wide variety of samples including animal feaces, manure and soil (Pitta et al., 2016). However, the overuse of antibiotics for growth promotion or prophylaxis has contributed to an increased dissemination of antibiotic resistance genes among bacteria, including major human pathogens leading to widespread antibiotic resistance (Casewell et al., 2003). Collections of antibiotic resistant genes, often referred to as the resistome, were uncovered in reservoirs, such as human, animals, food and the environment (Rolain, 2013). Genes in resistomes are mobilizable and can therefore be transferred to human pathogens, generating drug resistant strains. NGS-based techniques played an important role in demonstrating the transferable nature of resistome-related genes. Initially a study using high capacity qPCR observed a co-enrichment of antibiotic resistance genes and transposons demonstrating the mobility of antibiotic resistant genes (Zhu et al., 2013). A resistome comprising genes active against 18 antimicrobial classes was observed across all samples. Metagenomics was also used to study the effect of antibiotic treatment on swine microbiota and demonstrated that there was a shift in the bacterial phylotypes driven by an increase in *Escherichia coli* populations 14 days post treatment (Looft et al., 2012). Along with an increase in the abundance of antibiotic resistance-encoding genes, the up-regulation of genes encoding aminoglycoside O-phosphotransferases showed that antibiotic treatment of farm animals promoted cross-resistance.

## Considerations to improve the current NGS based food microbiome studies

NGS methods are highly efficient for microbiome related studies but there are still challenges and limitations to consider when applying these techniques to specific cases on food and food-related environments.

### Quality and quantity of recovered nucleic acid

Natural environment samples, such as soil and water, along with stool and saliva, and fermented food samples, such as cheese and kimchi, contain high numbers of microorganisms. Consequently, these samples can offer sufficient template nucleic acid for subsequent analysis. In contrast, sanitary control and maintenance of strict food production environment standards have made isolation of total DNA from these environments challenging (Anvarian et al., 2016). Frequent exposure to detergents from routine cleaning can cause injury to microorganisms colonizing this niche, thereby compromising the ability to recover sufficient template nucleic acid for analysis. Moreover, metal ions (Bickley et al., 1996), lipids and proteins (Rossen et al., 1992), detergent residues (Schrader et al., 2012), are all likely to inhibit nucleic acid isolation (Ahn et al., 2014). Further these are likely to inhibit downstream experimental procedures, leading to the recovery of low quality nucleic acid or PCR reaction failure. These inhibitors can be difficult to remove and this problem is compounded especially when samples contain a low bacterial load. Since an amplification step is involved, 16S rDNA approach is well suited to analyze the microbiome from such samples (Anvarian et al., 2016).

Different enrichment methods can be carried out to increase nucleic acid concentrations in samples. Prior to nucleic acid purification, selective or non-selective cultivation based pre-enrichment or enrichment can be used to generate microbiomes with higher target bacterial counts (Duan et al., 2009; Leonard et al., 2015). After nucleic acid purification and before downstream sequencing, whole genome amplification can be carried out to linearly amplify genomic material (Duan et al., 2009). However, these approaches may lead to amplification bias giving rise to changes in the microbiome composition (Kim and Bae, 2011). Nonetheless, it provides genomic DNA in sufficient quantity for subsequent sequencing. Total RNA is also able to be amplified using a similar whole transcriptome amplification strategy (Wu et al., 2011).

### Utilization of adequate controls during sequencing

Internal controls are essential in multi-omic strategies. Often these are never considered. A sequencing control should be designed to contain DNA sequences from known bacterial species, processed in parallel with other samples during sequencing, which could finally provide an estimation of the sequencing errors during downstream bioinformatics analysis. An ideal positive control could contain DNA isolated from mixture of multiple reference strains constituting a *mock community* either from NCBI (National Center for Biotechnology Information) and/or NCTC (National Collection of Type Cultures) collections (Kozich et al., 2013; Schloss et al., 2015). Strains closely related to the target of interest, such as lactic acid bacteria strains for fermented food microbiome studies (Humblot and Guyot, 2009), or a single strain as in *Staphylococcus aureus* DNA as in a gut microbiome study (Bull-Otterson et al., 2013) should be used. Ideally, a positive control should be applied in all 16S rDNA sequencing studies due to error associated with sequencing accuracy.

As with the positive, a negative control is also relevant in sequencing runs. Being high throughput, conventional amplicon based or whole genome sequencing experiments can analyze DNA at nanomolar concentrations. Trace levels of contamination present in the reagents used during DNA isolation or library preparation can introduce an undesirable bias in the analysis. This may interfere with final sequencing results giving rise to background contamination noise, especially when the target contains very few microorganisms (Salter et al., 2014). Water as template for PCR amplification is generally included as negative control in NGS experiments, while the usage of a no template control during 16S rDNA PCR is also common (Park et al., 2012). Currently few food related 16S rDNA sequencing studies take negative controls into consideration (Claesson et al., 2010; Wu et al., 2011; Park et al., 2012; Bull-Otterson et al., 2013; Burns et al., 2015; Burke and Darling, 2016), and most of these use negative controls applied only in during the PCR step (Salter et al., 2014).

## Conclusions and future perspectives

The utility of culture dependent approaches in the study of bacterial diversity is inherently limited in sensitivity. Nucleic acid sequencing platforms are being increasingly used to characterize bacteria and to identify bacterial communities from complex environmental matrices. With respect to food, popular sequencing technologies used to identify microbial communities include 16S rDNA sequencing, metagenomics and metatranscriptomics. 16S rDNA sequencing is currently the most commonly applied technique, while metagenomics and metatranscriptomics approaches are still underutilized to date. The latter approaches provide deep insights into the compositional and functional characteristics of a fermented food microbiome. Compared to fermented food, these technologies are rarely used to characterize the non-fermented food microbiome. In addition to discussing different sequencing chemistries currently available, this review has provided an overview of non-fermented food-related microbiome studies based on these next generation approaches. Overall, the potential benefits of multi-omic approaches in improving food safety was emphasized.

Even though, most metagenomic approaches use NGS platforms to sequence DNA, third generation sequencing (TGS) strategies are not routinely used for microbiome studies. Recently, a third generation sequencing MinION platform was used to sequence full length 16S rDNA amplicons generated from a synthetic community of ten microbial species with varying relative abundance (Li et al., 2016). The MinION based whole 16S rDNA amplicon profiling identified species correctly and the abundance profiles correlated with defined abundances. The latter technique offers some improvements in terms of the depth of data obtained when compared to the current 16S rDNA strategies by allowing species level characterization of the microbes. Soon it can be expected that TGS platforms will gain wider applications related to food production. In time, these data can be carefully analyzed to design new techniques to enhance process efficiency and product quality.

## Author contributions

YC, SF, and SS organized the draft and wrote the manuscript. YC, SF, SP, KJ, and SS participated in the critical revision of the manuscript. All authors read and approved the manuscript.

### Conflict of interest statement

The authors declare that the research was conducted in the absence of any commercial or financial relationships that could be construed as a potential conflict of interest.
